# Depopulation of Caged Layer Hens with a Compressed Air Foam System

**DOI:** 10.3390/ani8010011

**Published:** 2018-01-11

**Authors:** Shailesh Gurung, John Hoffman, Kendre Stringfellow, Daad Abi-Ghanem, Dan Zhao, David Caldwell, Jason Lee, Darrel Styles, Luc Berghman, James Byrd, Yuhua Farnell, Gregory Archer, Morgan Farnell

**Affiliations:** 1 Department of Poultry Science, Texas A&M AgriLife Research and Extension, College Station, TX 77843, USA; sg45902@tamu.edu (S.G.); john.hoffman@cobb-vantress.com (J.H.); kendre.stringfellow@abvista.com (K.S.); daad.abighanem@gmail.com (D.A.-G.); dz137@tamu.edu (D.Z.); caldwell@tamu.edu (D.C.); jtlee@tamu.edu (J.L.); berghman@poultry.tamu.edu (L.B.); yfarnell@tamu.edu (Y.F.); garcher@tamu.edu (G.A.); 2 Veterinary Services, Animal and Plant Health Inspection Service- US Department of Agriculture, Riverdale Park, MD 20737, USA; darrel.k.styles@aphis.usda.gov; 3 Southern Plains Agricultural Research Center, Agricultural Research Service, US Department of Agriculture, College Station, TX 77843, USA; byrdmen8@yahoo.com

**Keywords:** HPAI, depopulation, compressed air foam, caged layers

## Abstract

**Simple Summary:**

Reportable diseases, such as avian influenza, spread rapidly among poultry, resulting in the death of a large number of birds. Once such a disease has been diagnosed at a farm, infected and susceptible birds are rapidly killed to prevent the spread of the disease. The methods to eliminate infected caged laying hens are limited. An experiment was conducted to study the effectiveness of foam made from compressed air, water, and soap to kill laying hens in cages. The study found that stress levels of the hens killed using compressed air foam in cages to be similar to the hens killed by carbon dioxide or the negative control. Hens exposed to carbon dioxide died earlier as compared to the foam methods. The authors conclude that application of compressed air foam in cages is an alternative to methods such as gas inhalation and ventilation shutdown to rapidly and humanely kill laying hens during epidemics.

**Abstract:**

During the 2014–2015 US highly pathogenic avian influenza (HPAI) outbreak, 50.4 million commercial layers and turkeys were affected, resulting in economic losses of $3.3 billion. Rapid depopulation of infected poultry is vital to contain and eradicate reportable diseases like HPAI. The hypothesis of the experiment was that a compressed air foam (CAF) system may be used as an alternative to carbon dioxide (CO_2_) inhalation for depopulating caged layer hens. The objective of this study was to evaluate corticosterone (CORT) and time to cessation of movement (COM) of hens subjected to CAF, CO_2_ inhalation, and negative control (NEG) treatments. In Experiment 1, two independent trials were conducted using young and spent hens. Experiment 1 consisted of five treatments: NEG, CO_2_ added to a chamber, a CO_2_ pre-charged chamber, CAF in cages, and CAF in a chamber. In Experiment 2, only spent hens were randomly assigned to three treatments: CAF in cages, CO_2_ added to a chamber, and aspirated foam. Serum CORT levels of young hens were not significantly different among the CAF in cages, CAF in a chamber, NEG control, and CO_2_ inhalation treatments. However, spent hens subjected to the CAF in a chamber had significantly higher CORT levels than birds in the rest of the treatments. Times to COM of spent hens subjected to CAF in cages and aspirated foam were significantly greater than of birds exposed to the CO_2_ in a chamber treatment. These data suggest that applying CAF in cages is a viable alternative for layer hen depopulation during a reportable disease outbreak.

## 1. Introduction

The US poultry industry has faced disease outbreaks and natural disasters that require flocks to be destroyed. Natural disasters such as hurricanes and floods cause damage to poultry houses, feed mills, roads, and power lines leading to emergency killing of flocks to prevent further suffering [1]. Reportable poultry diseases such as highly pathogenic avian influenza (HPAI), exotic Newcastle disease (END), avian infectious laryngotracheitis, avian infectious bronchitis, and mycoplasmosis are also threats to the poultry industry [2]. The last reported US END outbreak was in 2002–2003 in California, which resulted in the loss of 3.16 million birds and $161 million [3]. Most recently, the US poultry industry lost 43 million layer and pullet hens and 7.4 million turkeys during the 2014–2015 HPAI outbreak [4]. The layers and turkeys lost alone were worth $1.6 billion, and the overall economic losses were estimated to be $3.3 billion [5,6].

Euthanasia is the act of terminating the life of an animal in a way that involves minimum pain and distress [7]. Euthanasia, meaning a good death, is distinct from depopulation. On the other hand, depopulation is an emergency measure used to rapidly kill animals with as much consideration given to their welfare as possible [7,8]. Elimination of poultry infected or at risk of infection from HPAI is a primary US animal health policy to control and eradicate the virus [9]. Therefore, birds within a three kilometer radius of an infected zone are killed and disposed of [10]. Methods used for mass depopulation of poultry depend upon the species, housing type, age of bird, ambient temperature, and available carcass disposal methods [1].

The American Veterinary Medical Association (AVMA) has approved carbon dioxide (CO_2_) inhalation as a means of euthanizing poultry [7]. Carbon dioxide is an analgesic and anesthetic gas [11,12]. Exposure to CO_2_ inhalation induces hypercapnic hypoxia in birds, which results in rapid unconsciousness and ultimately leads to death [13]. However, mammals and birds may show aversive responses to CO_2_ inhalation [14]. Humans exposed to CO_2_ concentrations between 40% and 55% experienced painful sensations [15] due to acidification of respiratory mucosa upon exposure to CO_2_ [16]. Birds experience respiratory distress such as gasping (breathlessness) and deep breathing while remaining conscious [17]. Birds exposed to liquid CO_2_ may also suffer from cold stress [18]. The use of CO_2_ inhalation may not be suitable for some types of poultry houses due to differences in construction. The method requires effective sealing of poultry houses, special equipment, and the rapid flow of a large volume of gas over the birds [1,17]. Application of CO_2_ also presents a safety risk to human personnel involved [17].

Water-based foam is a suitable depopulation alternative to CO_2_ inhalation of floor reared poultry [8]. The USDA Animal and Plant Health Inspection Service (APHIS) and the AVMA have accepted the use of water-based foam for killing commercial broilers and turkeys (poultry that are not housed in cages) [8]. Aspirated foam nozzles and high-expansion foam generator systems produce the water-based foam used for floor-reared poultry [19,20]. The USDA-APHIS performance standard recommends the bubble size of the water-based foam to be 1.58 cm (0.625 inches) or less. The water content of the foam should be enough that allows it to flow around the structures and at the same time cover the birds on the floor [8]. Foam is a collection of air-filled bubbles derived from a solution of detergents and water [21]. Foam has been widely used by firefighting departments for extinguishing fires [22,23]. Water-based foam depopulation was developed in response to the 2004 Delmarva avian influenza (AI) event [1]. Broilers and turkeys are immersed in foam which blocks the respiratory tract resulting in hypoxia, leading to loss of consciousness, convulsions, cerebral death, and cessation of cardiac activity [24]. However, depopulation of caged layer hens poses a different challenge. Foam developed for floor-reared poultry is a wet foam that drains quickly through mesh cage floors, making it unfit for caged layer houses. Furthermore, commercial cage farms have high stocking densities (100,000 or more layers per house) and are multi-tier buildings (5–10 tiers of cages), which limit access to conventional foaming methods [25]. The outbreak of a disease, like AI, in a caged layer facility would be the worst-case scenario, as a large number of birds would have to be depopulated rapidly, safely, and as humanely as possible.

The methods used for mass depopulation should be efficient and give due consideration to personnel safety and bird welfare. Birds subjected to euthanasia and depopulation methods are likely to suffer stress until they lose consciousness. It is vital for a depopulation method to result in a quick death to minimize suffering and contain the virus. Birds undergo clonic and tonic convulsions upon loss of consciousness during euthanasia [26,27]. Termination of such neuromuscular spasms (or cessation of movement) is an indicator of brain death [28]. Birds engulfed by foam cannot be visually evaluated for behavioral changes such as loss of posture or onset and cessation of convulsions. Accelerometers are sensors that measure changes in velocity due to movement [29]. Dawson and colleagues [27,30] determined time to cessation of movement of broilers subjected to water-based foam depopulation based on their accelerometer data. Physiological responses of birds to stress are mediated by the limbic hypothalamo-pituitary-adrenocortical (HPA) axis and sympathetic-adrenalmedullary (SAM) axis [31]. The effects of HPA axis are mediated by release of glucocorticoids, like corticosterone, in response to stress [32,33]. Serum corticosterone is a common physiological parameter used to assess welfare in birds [34,35,36,37].

Aspirated and high expansion foams, used to depopulate floor reared poultry, are made by utilizing the kinetic energy from the flow of water. On the other hand, a compressed air foam (CAF) system uses energy from compressed air. Expansion ratio is a foam quality parameter. It is defined as the ratio of volume of finished foam to volume of aqueous foam solution [38]. Foam is classified into three kinds: low, medium, and high expansion foam with expansion ratios less than 20:1, 20:1 to 200:1, and more than 200:1, respectively [39]. Aspirated foam has a medium expansion ratio, while compressed air foam ranges from low to medium expansion. Since a CAF system is a closed system, foam concentrate, water, and air can be manipulated as required to produce finished foam. Benson and colleagues [1] were first to report the application of CAF for depopulation of broilers. A CAF system consists of a centrifugal water pump, a foam concentrate proportioner, an air compressor, and a mixing chamber [21,40]. In a CAF system, compressed air agitates a foam solution in a mixing chamber to produce a finished foam [40]. The ratio of aqueous foam solution and compressed air can be adjusted in a CAF system to produce a drier or wetter foam [23,41]. Ideal foam for cage operations (conventional, colony, or enriched colony) would be one that has a longer dewatering time and a small bubble size, since such foam would persist long enough in cages depriving birds of oxygen ultimately leading to death from hypoxia due to occlusion of the trachea. Compressed air foam has a uniform bubble size and better stability than air-aspirated foams [23].

The hypothesis of the experiment was that a compressed air foam system is a rapid and humane means for caged layer hen depopulation. The specific objectives of this study were to determine and compare serum corticosterone levels and time to cessation of movement of birds subjected to CAF, CO_2_ inhalation, and negative control treatments. Hens subjected to CAF and CO_2_ treatments were necropsied to evaluate signs of trauma or presence of foam in the respiratory tract.

## 2. Materials and Methods

### 2.1. Experimental Animals

Lohmann LSL young and spent hens were obtained from an egg integrator and housed in the Texas A&M University Poultry Science Research, Teaching, and Extension Center layer barn prior to the experiments. The young hens were 20 weeks of age while the spent hens were 76–95 weeks old. All birds were cared for under an approved TAMU Institutional Animal Care and Use Committee (IACUC) protocol # 2009-222.

### 2.2. Experiments

Based on the objectives of the study, two separate experiments were carried out. The objective of Experiment 1 was to evaluate corticosterone (CORT) levels, while that of Experiment 2 was to determine time to cessation of movement (COM). Young and spent hens, separately, were the test subjects in Experiment 1, while only spent hens were used in Experiment 2. Birds in Experiment 1 were subjected to five treatments: negative control (NEG), CO_2_ added to a chamber after bird placement, pre-charged CO_2_ chamber, CAF in cages, and CAF in a chamber. Treatments were performed on two replicate days for each age group for a total of four replications for Experiment 1. Twelve (*N* = 6 and 6) young hens and 13 (*N* = 6 and 7) spent hens were subjected to each of the five treatments. Experiment 2 consisted of three treatments: CO_2_ added to a chamber, CAF in cages, and aspirated foam in a floor pen on the same day. The number of spent hens randomly assigned to each of the three treatments in Experiment 2 was 16. The concentration of CO_2_ in both experiments was 100%. A 208 L plastic chamber with a floor area of 3561 cm^2^ and height of 84 cm was used for application of treatments in both experiments. The hens in the NEG treatment were placed in cages and briefly restrained for jugular venipuncture. Compressed air foam was applied in the CAF in cages and CAF in a chamber treatments. The dimensions of each A-frame pullet cage was 0.61 m × 0.57 m × 0.38 m. Three to four hens were placed at a time in each chamber and cage prior to treatments. In Experiment 2, a group of 16 hens allocated to the aspirated foam treatment were placed in a floor pen of the dimensions of 2.44 m × 2.44 m The conventional cages used in the CAF in cages treatment, in Experiments 1 and 2, were positioned 0.1524 m above a plywood platform to simulate a layer house manure belt. Compressed gas tanks of 100% CO_2_ were purchased from a local supplier. The flow rate of the gas for the CO_2_ added to a chamber treatment was set at 62 L per minute to achieve a gas displacement rate of 30% of the chamber volume in a minute. The chamber was filled for three minutes after hens were placed inside it. The same flow rate and time period was followed in the pre-charged CO_2_ chamber treatment. The hens were placed inside the chamber after the end of three minutes. However, gas concentration was not measured in the chamber.

### 2.3. Foam Production

The CAF unit (Rowe CAF LLC, Hope, AR, USA) consisted of a 1982 L per minute rotary screw air compressor (Vanair Inc., Michigan City, IN, USA), a 40.55 metric horsepower gasoline engine (Kohler Co., Kohler, WI, USA), a 567 L per minute centrifugal water pump (Hale Products, Inc., Ocala, FL, USA), and a foam concentrate proportioner (0.1%–10%; FoamPro, Kingston, NY, USA). Water for the CAF was supplied from a 1135.6 L tank. A 37.9 L foam cell contained the foam concentrate necessary for the experiment. A Class A foam concentrate (ICL Performance Products, Rancho Cucamonga, CA, USA) was used at 3.5% in the CAF and 1% in the aspirated foam. Foam concentrate was injected by the proportioner into the water manifold of the CAF unit. A separate air manifold supplied compressed air to the mixing chamber. These three constituents of foam were agitated in the mixing chamber of the CAF unit. The flow rates of air and water into the mixing chamber were adjusted to produce a foam of the desired consistency and thickness. The final foam had consistency of that of a shaving cream (on visual inspection). Flow rate of aqueous foam solution and volume of compressed air are key to producing finished foam of desired nature. The compressed air foam produced was thick enough to stay in the cage for 5 min or longer and had a mean expansion ratio of 111:1. The finished CAF was released from a single 3.8 cm CAF unit through a 15 m long and 3.8 cm internal diameter firefighting hose attached to a 6.4 cm wide and 6 m long suction hose. This significantly reduced the velocity of the finished foam minimizing the impact on the hens. The foam stream was initially directed at the floor of the cage or chamber in consideration for the birds’ welfare. The flow rate of CAF produced during this study was 1874 L per minute. Foam was applied to the cage for two minutes or filled the chamber within one minute. In Experiment 2, an aspirated nozzle (Spumifer American Company, Ridgefield Park, NJ, USA) was used to produce aspirated foam, a method conditionally approved for depopulation of floor-reared broilers and turkeys. The aspirated foam system had a flow rate of an estimated 11,356 L per minute. Environmental factors such as temperature and humidity may affect foam quality. However, all five treatments were performed on the same day, at the same location, with the same settings and same equipment, within an enclosed poultry barn.

### 2.4. Experiment 1

Serum CORT levels of young and spent hens were evaluated separately. A total of 65 spent hens and 60 young hens were used. The hens were subjected to foam, CO_2_, and NEG control for four minutes, after which they were removed. Death was ascertained by observing corneal and pedal reflexes of the birds. Blood samples were collected immediately (within 2 min) by severing the femoral artery of hens in all treatments except the NEG control. The birds in the NEG control group were alive until venipuncture of the jugular vein for blood collection. These birds were euthanized afterward by cervical dislocation. Blood was allowed to clot for 1 h at room temperature and transported on ice to the laboratory. The blood was stored at 4 °C for 24 h. Serum was collected by centrifuging blood at 1000× *g* for 15 min. Serum corticosterone was evaluated by competitive ELISA kit ADI-901-097 (ENZO Life Sciences, Farmingdale, NY, USA) according to the instructions from the manual. Samples were run in duplicate. Absorbance was measured for each sample using a plate reader at 405 nm (BioTek Instruments Inc., Winooski, VT, USA). A four-parameter logistic regression model was used for curve fitting to interpolate the CORT levels from absorbance readings for each individual experimental subject. Intra-assay and inter-assay variability were 2.8% and 7.2%, respectively.

### 2.5. Experiment 2

The experiment was conducted to determine time to cessation of movement of laying hens subjected to each depopulation treatment. Accelerometer data loggers (HOBO Pendant G, Onset Computer Corporation, Bourne, MA, USA) were attached to the shanks of each bird by zip ties to measure changes in the movement as hens were exposed to the treatments. The accelerometers were programmed to start logging data every second. The end point of convulsive movements was determined as the point where a flat line (no signal) was recorded. The time-interval between start of treatment until loss of motion was determined for each hen. Forty-eight spent hens were used in total in this trial.

### 2.6. Postmortem Examination

Gross necropsy was performed on hens subjected to foam or CO_2_ treatments in Experiment 1. Respiratory tracts were visually evaluated for signs of physical injury or presence of foam.

### 2.7. Statistical Analysis

In Experiment 1, samples with concentrations outside the range of the standard curve were removed from the study. Therefore in the case of pullet hens, four samples were removed from the CO_2_ in cage group and one sample was omitted from the NEG control group. In the case of spent hens, one sample from the CAF in a chamber group was removed. Except for the CAF in a chamber group, which had 12 samples, each of the remaining four treatments had 13 samples. Statistical analyses were performed on CORT values of serum samples from a total of 55 young and 64 spent hens. Tests for Normality (Shapiro-Wilk) and Levene’s test for homogeneity of variance were performed on CORT and COM data using the PROC Univariate and PROC ANOVA procedures, respectively. Statistical analyses of CORT and time to COM data were done by one-way ANOVA using the PROC ANOVA procedure (SAS 9.4, Cary, NC, USA). Means deemed significant were further evaluated using Fisher’s LSD post-hoc test. The tests were carried out at the 5% level of significance (α = 0.05).

## 3. Results and Discussion

### 3.1. Weather Conditions

In Experiment 1, the temperature and relative humidity, during the young hen study, were 18.9 °C and 89% on the first replicate day and 25 °C and 83% on the second replicate day. Similarly for the spent hen study, the temperature and relative humidity data were 24.4 °C and 87% on the first replicate day and 27.8 °C and 29% on the second replicate day. In Experiment 2, the temperature and relative humidity were 28.9 °C and 72%, respectively.

### 3.2. Serum Corticosterone Concentrations

**Figure 1:**
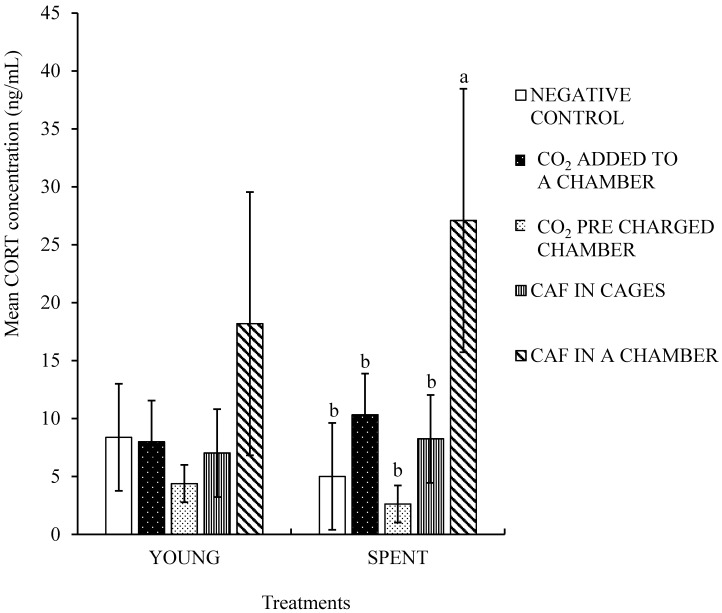
Corticosterone concentration of young and spent hens. The corticosterone (CORT) concentrations were measured in duplicates and expressed in ng/mL. Bars (mean ± SEM) with different superscripts are statistically significantly different by Fisher’s LSD test (*p* < 0.05). Young and spent hen trials were conducted separately. Twelve young hens and 13 spent hens were used in each treatment.

All birds subjected to CO_2_ or foam treatments died. None of the hens (young or spent) survived. The mean serum CORT concentration of young hens subjected to the NEG control, CO_2_ added to a chamber, CO_2_ pre-charged chamber, CAF in cages, and CAF in a chamber treatments were 8.4, 8.0, 4.4, 7.0, and 18.2 ng/mL, respectively (Figure 1). There were no statistically significant differences in serum CORT concentration of young hens subjected to the five treatments (*p =* 0.569). The stress responses of the young hens to gas inhalation and compressed air foam treatments were comparable to the NEG control group. Unlike the young hens, a statistically significant difference was observed in the CORT concentrations of spent hens among the treatment groups (*p* = 0.0005). Birds in the CAF in a chamber group had significantly higher CORT levels than hens in the remaining four treatment groups. The mean serum CORT concentrations of spent hens assigned to CAF in a chamber group was 27.1 ng/mL while that of the NEG control, CO_2_ added to a chamber, CO_2_ pre-charged chamber, and CAF in cages were 5.0, 10.3, 2.6, and 8.2 ng/mL, respectively. Though only numerically different, the CORT level of young hens subjected to CAF in a chamber was higher than rest of the four treatments. Yan et al. [42] reported no age-related effects on CORT levels of caged White Leghorn pullets.

Scanes [43] reported that stressors like heat, cold, floor space, restraining, catching, shackling, feed restriction, and nutrient deficiency elevates plasma CORT in poultry. Exposure to CO_2_ has also been reported to cause pain or distress in animals [44,45]. The results of Experiment 1 demonstrates that the CAF in a chamber method was significantly more stressful to spent hens than the CO_2_ inhalation, CAF in cages, and NEG control treatments. The chamber was a novel environment to the hens, which might have resulted in higher CORT levels than the CAF in a cage or NEG control treatment. However, the serum CORT levels of young hens as well as spent hens subjected to CAF in cages were similar to the NEG control and CO_2_ inhalation treatments. Benson and colleagues [1] reported that serum CORT levels of broilers had no statistically significant difference among compressed air foam alone, compressed air foam with CO_2_, and the CO_2_ polyethylene tent method. Hens subjected to the pre-charged CO_2_ chamber had numerically lower CORT levels as compared to the other treatments. It is likely that these hens lost consciousness earlier than hens in other treatments. CO_2_ reduces the intracellular brain pH, leading to induction of anesthesia [46]. The hens were subjected to higher concentration of CO_2_ at once in this treatment.

### 3.3. Time to Cessation of Movement

**Figure 2:**
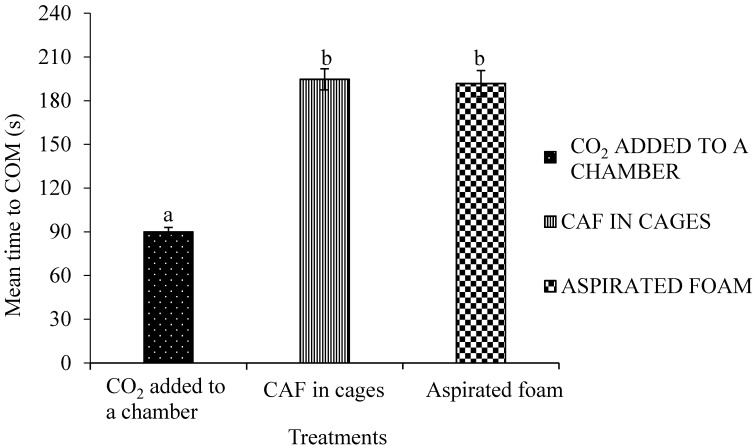
Time to cessation of movement (±SEM) of spent hens. Bars (mean ± SEM) with different superscripts have statistically significant difference by Fisher’s LSD test (*p* < 0.05). Sixteen spent hens were randomly assigned to each treatment. The treatments in this trial were carbon dioxide added to a chamber, compressed air foam in cages, and aspirated foam.

Birds undergo terminal convulsive movements after onset of unconsciousness until they become motionless [27]. The termination of clonic and tonic phase of convulsions is known as cessation of movement. The times to COM (or cessation time) of spent hens subjected to the CAF in cages, CO_2_ added to a chamber and aspirated foam in floor pens were determined based on accelerometer readings (Figure 2). The average time to COM was 90 s for birds in CO_2_ in a chamber, 195 s for birds in CAF in cages, and 192 s for hens subjected to aspirated foam treatments. A statistically significant difference in mean time to COM was observed among the three treatments (*p* < 0.05). A post hoc Fisher’s LSD test revealed that time to cessation of movement of hens subjected to CO_2_ added to a chamber was significantly shorter than that of hens in the CAF in cages and aspirated foam groups.

In the present study, times to cessation of movement of spent hens assigned to CAF in cages and aspirated foam treatments were within the ranges reported in previous studies. Based on electrocardiography (ECG) readings, Benson et al. [1] reported that time to cessation of cardiac activity of broilers subjected to foam with CO_2_, foam without CO_2_, and the polyethylene tent method were 73 s, 64 s, and 139 s, respectively. Dawson et al. [27] reported the time to cessation of movement of broilers subjected to water-based foam to be in range from 25 to 179 s. In this study, spent hens depopulated using CO_2_ took significantly less time to be motionless than hens subjected to the aspirated foam and CAF in cages. Loss of consciousness is followed by onset of convulsions [7]. A method that results in quicker loss of consciousness might lead to earlier onset of convulsions, resulting in a shorter time to COM. In this study, the hens in the CO_2_ added to a chamber group had shorter time to COM, which might suggest that those birds lost consciousness earlier than the hens in other treatments. Mean cessation times of spent hens belonging to the CAF in cages and aspirated foam treatments were not significantly different. Wing flapping and struggling of the hens may have agitated the foam bubbles creating air pockets in the foam treatments. The time taken for foam to fill the cages and form a blanket of foam around the birds was longer than filling of the chamber with CO_2_, which may suggest a second reason for increased cessation times in both of the foam treatments.

### 3.4. Gross Necropsy Findings

Gross necropsy was performed on hens subjected to CAF and CO_2_ treatments in Experiment 1. The trachea, syrinx, and bronchi were evaluated for signs of hemorrhage, presence of foam, and presence of blood. Traces of foam were present in the upper trachea (first 5 cm) but absent in the lower trachea, syrinx, and bronchi. There were no signs of trauma or injury in the airways of hens since no blood or hemorrhages were found on necropsy. The hens randomly assigned to the CO_2_ added to a chamber and CO_2_ pre-charged chamber groups had no signs of hemorrhage or injury to the respiratory tract. Exposure to CO_2_ treatments resulted in asphyxiation followed by generalized hypoxia and death. The cause of death of all hens subjected to the CAF treatments was due to occlusion of airways by foam leading to hypoxia. Benson et al. [1] reported that broilers subjected to water-based foam died due to hypoxia due to occlusion of the trachea. In the same study, Benson and colleagues showed that foam was present in the trachea and lungs of broiler chickens. Blood was found in the syrinx, bronchi, and lungs upon histological examination. Raj et al. [47] conducted a trial to kill end-of-lay hens using a dry nitrogen foam in which they observed foam bubbles around the larynx and upper part of trachea. McKeegan et al. [17] in their study on gas-filled high-expansion foam observed small foam bubbles in trachea and tracheal openings of broilers exposed to high expansion nitrogen foam.

The use of CAF for depopulation does not require manual handling of live birds. Unlike CO_2_ inhalation, CAF does not present safety risks to the personnel involved. In addition, a CAF can be used as a means for cleaning and disinfection of infected premises after completion of depopulation and disposal of the carcasses [48]. This paper is the first peer-reviewed manuscript on the application of CAF as a means of depopulation of caged layer hens.

## 4. Conclusions

The CORT levels of hens subjected to CAF in cages and the AVMA approved CO_2_ inhalation treatments were similar to that of birds in the NEG control. The use of CO_2_ elicited COM earlier than foam-based treatments, possibly due to the anesthetic properties of the gas and differences in filling times of the two methods. The presence of foam in the upper trachea of hens confirms that the cause of death was due to occlusion of the trachea leading up to hypoxia. These findings suggest that the application of compressed air foam in cages may be a viable means for depopulation of caged layer hens during reportable disease outbreaks or natural disasters. Further research needs to be conducted on the addition of carbon dioxide and nitrogen-infused CAF for layer hen depopulation.
